# Socio-economic determinants for the place of last care: results from the acute palliative care unit of a large comprehensive cancer center in a high-income country in Europe

**DOI:** 10.1186/s12904-023-01240-2

**Published:** 2023-08-08

**Authors:** Sebastian M. Christ, Ellen Hünerwadel, Bigna Hut, Maiwand Ahmadsei, Oliver Matthes, Annina Seiler, Markus Schettle, David Blum, Caroline Hertler

**Affiliations:** 1 Department of Radiation Oncology, University Hospital Zurich, University of Zurich, Zurich, Switzerland; 2 Faculty of Medicine, University of Zurich, Zurich, Switzerland; 3 Department of Consultant Psychiatry and Psychosomatic Medicine, University Hospital Zurich, University of Zurich, Zurich, Switzerland; 4 Competence Center Palliative Care, Department of Radiation Oncology, University Hospital Zurich and University of Zurich, Zurich, Switzerland

**Keywords:** Palliative care, Place of last care, Socio-economic determinants

## Abstract

**Background and introduction:**

The place of last care carries importance for patients at the end of life. It is influenced by the realities of the social welfare and healthcare systems, cultural aspects, and symptom burden. This study aims to investigate the place of care trajectories of patients admitted to an acute palliative care unit.

**Materials and methods:**

The medical records of all patients hospitalized on our acute palliative care unit in 2019 were assessed. Demographic, socio-economic and disease characteristics were recorded. Descriptive and inferential statistics were used to identify determinants for place of last care.

**Results:**

A total of 377 patients were included in this study. Median age was 71 (IQR, 59–81) years. Of these patients, 56% (n = 210) were male. The majority of patients was Swiss (80%; n = 300); about 60% (n = 226) reported a Christian confession; and 77% had completed high school or tertiary education. Most patients (80%, n = 300) had a cancer diagnosis. The acute palliative care unit was the place of last care for 54% of patients. Gender, nationality, religion, health insurance, and highest level of completed education were no predictors for place of last care, yet previous outpatient palliative care involvement decreased the odds of dying in a hospital (OR, 0.301; 95% CI, 0.180–0.505; p-value < 0.001).

**Conclusion:**

More than half of patients admitted for end-of-life care died on the acute palliative care unit. While socio-economic factors did not determine place of last care, previous involvement of outpatient palliative care is a lever to facilitate dying at home.

**Supplementary Information:**

The online version contains supplementary material available at 10.1186/s12904-023-01240-2.

## Introduction and background

Place of last care (PLC) is an important aspect of end-of-life (EoL) care. Catering to patients’ preferences with respect to PLC is increasingly regarded as a quality marker for EoL care. PLC is influenced by many factors, including the structure of the social welfare and healthcare systems, cultural and personal beliefs and preferences, as well as the clinical disease reality with its associated symptoms. In high-income countries, the majority of people would like to die at home. Yet a large proportion of patients continues to die in hospitals and long-term care facilities [1, 2]. This is in contrast to many low- and middle-income countries, where a larger proportion of patients is estimated to die at home [3].

There have been various smaller studies and systematic reviews [4–6] examining PLC and identifying predictors for the PLC in different cohorts of palliative care patients. *Tay et al. (2021)* assessed 359 patients having received home-based palliative care in Singapore. The authors reported that 58% of patients died at home and found higher functional status, greater pain intensity and non-home death preference to be positively linked with an institution as LPC [7]. *Alawneh et al. (2020)* analyzed 630 patients who had a palliative care consultation at a tertiary cancer center in Jordan. A minority of 13% of patients died at home. Male gender, age greater than 65 years, earlier palliative care integration, and involvement of home care services were positively associated with dying at home [8]. In a cohort of 2,066 who had home-based palliative care in Canada, *Wales et al. (2019)* found that 48% died at home, and the authors identified the lowest income quintile as having an increased odds of a hospital ward being the LPC [9]. In an analysis based on national health insurance data from 2013 for France, 60% of 347,253 patients died in a hospital setting. The authors of this study did not conduct a regression analysis to identify determinants for PLC [1]. Even though some authors suggested that place of death is an imperfect indicator for the quality of care at EoL [10], PLC constitutes an important, yet under-investigated metric.

Switzerland is a country located in Europe with a size of about 41.3sqkm, a population of 8.7 m inhabitants, and the second largest GDP per capita worldwide with USD93k in 2021 [11, 12]. Healthcare in Switzerland is universal, largely paid for directly by its citizens, via basic health insurance coverage and out-of-pocket expenses. About 71k people died in Switzerland in 2021 [12]. Like in other European nations, the majority of the population has a preference to die at home [13]. Yet as recently as 2009, 41% of people died in a hospital setting and 40% in a long-term care facility, with only a share of the remainder of patients actually having died at home [13]. The share of patients who die inside hospitals in Switzerland has risen over the past years and decades [14]. To our knowledge, PLC and its predictors have not been systematically evaluated for different palliative patient populations in Switzerland to date. The aim of this study is therefore to investigate PLC and selected socio-economic determinants for the subgroup of patients who required hospitalization on the acute palliative care unit (APCU) of a large comprehensive cancer center (CCC) in Switzerland, which serves as an exemplary high-income country with a high-quality healthcare system in the heart of Europe.

## Materials and methods

### Study design

This study was conceptualized as a single-center observational cohort study, which was conducted at the Radiation Oncology Department of the University Hospital Zurich (USZ). The APCU of our department is integral part of the university hospital’s CCC, from which patients are referred. The APCU is also open to non-oncological patients.

### Patient population

Patients were included in this study if they (a) were adults, (b) were hospitalized on our APCU from January to December 2019, and (c) had provided general consent of the use of their data for scientific purposes.

### Study endpoints

The study was conceptualized to analyze PLC and its socio-economic determinants. PLC was defined as the location, where patients spent at least the last couple of days of their lives. Hospital, nursing facility, rehabilitation facility, hospice and home were chosen as PLC categories.

### Data collection process

Data on demographics, socio-economic and basic clinical parameters were manually extracted from the electronic medical records (EMR) KISIM™ by two independent researchers (EH and BH) and quality-checked by another researcher (SMC). PLC was categorized as specified above; for patients who were discharged from our APCU, the PLC was established thorough (1) a review of medical reports sent to our institution after the death of the patients, (2) active follow-up with family physicians, smaller hospitals or nursing homes, (3) getting into contact with family members. The following socio-economic variables were assessed: Gender, nationality, confession, health insurance plan, highest level of completed education, general living situation (urban/rural), social living situation pre-admission, and next relative. Selected factors pertaining to individual preferences or aspects of EoL care were also assessed. Predefined variables were available for the majority of patients.

### Statistical analysis

Manually extracted data was assembled in the spreadsheet program Microsoft© Excel© (version v.16). Descriptive summary statistics were computed for all variables under study. Uni- and multivariable logistic regression analysis was used to identify determinants for the PLC. Statistical significance was set at < 0.05. Statistical analysis was conducted by one researcher (SMC) and quality-checked by another researcher (CH). The statistical software package STATA (v16.1) was used to conduct all quantitative analysis.

### Ethical approval

This study was approved by the Swiss Cantonal Ethics Committee (BASEC ID #2019–02488). The authors made sure the study complied with the *World Medical Association International Code of Medical Ethics* and the STROBE checklist (see Supplements 1).

## Results

### Patient socio-economics

Median age of the 377 patients under study was 71 (interquartile range (IQR), 59–81). Forty-four (n = 167) percent of patients were female. The large majority (80%; n = 300) were Swiss. Slightly less than two thirds of patients (60%; n = 226) were of Christian faith. Almost 80% (n = 297) of patients had public health insurance. The highest level of completed education was high school or tertiary education (including colleges, universities, technical training institutes, and vocational schools) for 77% (n = 287) patients, while for 23% (n = 88) of patients, education status remained unknown. For 80% (n = 300) of patients, cancer was the primary diagnosis (Table 1).

**Table 1:** Basic patient characteristics

Variable	Data (n = 377 patients)
Age, median (IQR)	71 (59–81)
Female gender, n (%)	167 (44)
Nationality, n (%)	
► Switzerland	300 (80)
► Italy	17 (5)
► Germany	11 (3)
► Kosovo	5 (1)
► Other	44 (12)
Confession, n (%)	
► Roman Catholic	126 (33)
► None	106 (28)
► Protestant	100 (27)
► Muslim	18 (5)
► Jewish	1 (0.3)
► Other	26 (7)
Health insurance plan, n (%)	
► General/public	297 (78)
► Private supplementary	48 (13)
► Fully private	34 (9)
Highest level of completed education, n (%)	
► Primary school	1 (0.3)
► Middle school	1 (0.3)
► High school	202 (54)
► Tertiary education^1^	85 (23)
► Unknown	88 (23)
Primary diagnosis, n (%)	
► Cancer	300 (80)
► Trauma	62 (16)
► Other	15 (4)

*Abbreviations:* IQR = Interquartile range

^1^Includes colleges, universities, technical training institutes, and vocational schools.

### Living situation and place of last care

Median length of stay (LoS) on the palliative care wards for all patients was 11 (5–17) days. Half of the patients were admitted from inpatient wards (50%; (n = 190), about a quarter lived at home (26%; n = 98) prior to hospitalization, and about a fifth of patients (19%; n = 70) was admitted via the intensive care unit (ICU) or emergency department (ED). Before admission, two thirds of patients (n = 250) lived in an urban setting. Sixty-one (n = 229) percent of patients shared a household with relatives, while less than a third (27%; n = 102) of patients lived alone. The next relatives were the partner or a child in 60% (n = 227) and 19% (n = 72) of cases, respectively (Table 2).

**Table 2:** End-of-life care and last place of care characteristics

Variable	Data (n = 377 patients)
LoS on palliative care, median (IQR)	11 (5–17)
General living situation, n (%)	
► Urban setting	250 (66)
► Rural setting	127 (34)
Admission from, n (%)	
► Inpatient wards	190 (50)
► Home	98 (26)
► Intensive care unit	42 (11)
► Emergency department	28 (8)
► Institution^1^	19 (5)
Discharge to, n (%)	
► Death on PC wards	204 (54)
► Home	77 (20)
► Nursing facility	54 (14)
► Hospice	18 (5)
► Rehabilitation facility	18 (5)
► Other hospital	6 (2)
Living situation pre-admission	
► With relatives	229 (61)
► Alone	102 (27)
► Institution	43 (11)
► Other^2^	3 (1)
Next relative	
► Partner	227 (60)
► Child	72 (19)
► Other relative	52 (14)
► Friend	17 (5)
► None	9 (2)
GP actively involved in EoL care	
► Yes	279 (74)

*Abbreviations:* EoL = End-of-Life; GP = General Practioner; IQR = Interquartile range; LoS = Length of stay; PC = Palliative Care

^1^Includes nursing or rehabilitation homes and other long-term care facilities.

^2^Includes palliative care or other specialized care institutions.

### Palliative care integration

By nature of the study, all patients received inpatient specialist PC (100%; n = 377). Prior to hospital admission, the General Practioner (GP) was closely involved in the coordination or direct patient care in 74% (n = 279) of cases. Post-discharge, the outpatient specialist PC was employed in slightly more than a quarter of patients (27%; n = 103), while the outpatient home care service was utilized by 36% (n = 134) of all patients. 60% (n = 224) of patients had advanced care directives, 55% (n = 206) had named a patient representative. While the ICU status was affirmative for less than 10% of patients (9%; n = 32), 97% (n = 363) of patients had an affirmative do-not-resuscitate (DNR) status (Table 3).

**Table 3:** Palliative care involvement characteristics

Variable	Data (n = 377 patients)
Specialist inpatient PC	377 (100)
Outpatient specialized PC post-discharge	103 (27)
Outpatient home care service post-discharge	134 (36)
Advance directives	224 (60)
Patient representative	206 (55)
ICU status (“Yes”)	32 (9)
DNR status (“No”)	363 (97)

*Abbreviations:* DNR = Do not resuscitate; ICU = Intensive care unit; IQR = Interquartile range; PC = Palliative care

### Uni- and multivariable analysis

On univariable logistic regression analysis, ten variables were significantly associated with the inpatient setting being the PLC. Age older than or equal to 70 years (Odds ratio (OR), 0.521 (95% confidence interval (CI), 0.312–0.872; p-value < 0.05), LoS of 10 days or more (OR, 1.773; 95% CI, 1.058–2.972; p-value < 0.05), admission from ED or ICU (OR, 10.991; 95% CI, 2.630–45.931; p-value < 0.001), and cancer diagnosis (OR, 3.874; 95% CI, 1.617–9.282; p-value < 0.05) were positively associated with “hospital” as PLC. Additionally, a partner as next relative (OR, 0.580; 95% CI, 0.338–0.994; p-value < 0.05), outpatient PC involvement pre-admission (OR, 0.083; 0.047–0.149; p-value < 0.001), no outpatient home care involvement pre-admission (OR, 0.301; 95% CI, 0.180–0.505; p-value < 0.001), advance care directives (OR, 0.549; 95% CI, 0.320–0.942; <0.05), negative ICU status (OR, 0.215; 95% CI, 0.102–0.453; p-value < 0.001), and DNR order (OR, 0.036; 95% CI, 0.008–0.166; p-value < 0.001) were also positively associated with hospital as LPC. On multivariable logistic regression analysis, the effect of three variables persisted, namely admission from ED/ICU (OR, 24.565; 95% CI, 2.095– 288.023; p-value < 0.05), previous involvement of outpatient PC service (OR, 0.105; 95% CI, 0.052–0.213; p-value < 0.001), and DNR order (OR, 0.012; 95% CI, 0.000–0.171; p-value < 0.001) (Table 4).

**Table 4:** Uni- and multivariable analysis for “hospital death” predictors

Variable	Univariable		Multivariable	
	OR (95% CI)	p-value	OR (95% CI)	p-value
Age				
► < 70 vs. ≥70	0.521 (0.312–0.872)	**< 0.05**	0.668 (0.345– 1.295)	0.233
Gender				
► Male vs. female	0.929 (0.560–1.540)	0.776		
Insurance				
► Private^1^ vs. non-private	0.929 (0.503–1.720)	0.817		
Level of schooling ► Until high school vs. tertiary^2^	1.207 (0.731–1.992)	0.462		
Nationality				
► Swiss vs. non-Swiss	1.133 (0.617– 2.082)	0.687		
Religion				
► Christian vs. non-Christian	0.944 (0.565– 1.577)	0.826		
Length of stay on PC				
► ≤ 10 days vs. >10 days	1.773 (1.058–2.972)	**< 0.05**	1.022 (0.345–2.042)	0.952
Source department				
► ED/ICU vs. all other	10.991 (2.630–45.931)	**< 0.001**	24.565 (2.095– 288.023)	**< 0.05**
Primary diagnosis				
► Cancer vs. no cancer	3.874 (1.617–9.282)	**< 0.05**	0.960 (0.310– 2.976)	0.945
Living conditions pre-admission				
► Alone vs. not alone	0.986 (0.561–1.731)	0.962		
Next relative				
► Partner vs. other	0.580 (0.338–0.994)	**< 0.05**	1.146 (0.574– 2.286)	0.699
Outpatient PC pre-admission				
► Yes vs. no	0.083 (0.047–0.149)	**< 0.001**	0.105 (0.052–0.213)	**< 0.001**
Outpatient home care pre-admission				
► Yes vs. no	0.301 (0.180–0.505)	**< 0.001**	0.581 (0.286–1.179)	0.133
Advance care directives				
► Yes vs. no	0.549 (0.320– 0.942)	**< 0.05**	0.504 (0.248– 1.025)	0.059
Patient representative				
► Yes vs. no	0.770 (0.463– 1.281)	0.314		
ICU status				
► Yes vs. no	0.215 (0.102–0.453)	**< 0.001**	0.539 (0.127–2.286)	0.402
DNR status				
► Yes (“do resuscitate”) vs. no	0.036 (0.008–0.166)	**< 0.001**	0.012 (0.000–0.171)	**< 0.001**

*Abbreviations:* CI = Confidence interval; DNR = Do not resuscitate; ED = Emergency department; ICU = Intensive care unit;

LoS = Length of stay; OR = Odds ratio; PC = Palliative care.

^1^Includes fully private and private supplementary plans.

^2^Includes colleges, universities, technical training institutes, and vocational schools.

## Discussion

Despite a recent increase in the interest for the quality of EoL care, quality of care remains inherently hard to measure. While there have been efforts to identify EoL quality measures beyond place of death, it remains the most commonly used metric in high-income countries [10]. In analyzing alternative measures for the quality of EoL care, *Hoare et al.* (2022) point out that place of death by itself does not actually provide an assessment of the *quality* of care [10]. However, framing the discussion around PLC rather than place of death acknowledges that quality of EoL care is not only about the actual location of death (hospital, nursing facility, rehabilitation facility, hospice, home), but, and maybe more importantly, around the care provided at the respective locations. While it is indeed true that dying at home is not a priority for all patients [15], especially when they understand their disease well [16], and that good symptom control might be more important than place of death, there are also studies which found that patients who die at home have less unmet care needs [17]. Nowadays, the quality of the social welfare and healthcare systems in many high-income countries are such that a high-quality of EoL can be achieved in both the outpatient and inpatient setting. It is therefore striking that there remains a discrepancy between actual and preferred PLC in high-and middle-income countries across the globe: In Jordan, 13% of patients treated at a CCC died at home [8]; in Switzerland, more than 80% of citizens died inside institutions [13], and in France, an estimated 60% of the general population died in a hospital setting [1]. The first step to counter this trend is to correctly record patients’ preferences [18]. *Ali et al.* (2017) have shown that correctly identifying PLC increases the chances of making the preferred PLC the actual PLC [19]. At our APCU, for example, preferred PLC is not yet systematically recorded at admission. However, as this information is the basis for a comprehensive resource assessment and as patients rarely change their preference with respect to PLC [20], time should be set aside to discuss PLC with patients, in order to narrow the gap between preferred and actual PLC in a high-income resource-rich country.

In our palliative patient population, socio-economic factors such as gender, nationality, religion, health insurance plan, and highest level of completed education were no determinants for PLC. This is in contrast to findings from other studies, for example, from Canada or the USA. *Wales et al. (2019)* found that the lowest income quintile in a population of patients who received home-based palliative care in Canada was a determinant for death in the hospital setting [9]. *Prioleau et al.* (2016), in reporting on predictors of place of death of 183 patients in a home-based primary and palliative care program in New York City, white skin color and non-Medicaid insurance plans were amongst the determinants for dying outside of the hospital [21]. While universal healthcare coverage in Switzerland might help level out socio-economic differences in healthcare provision, there is also evidence in the literature that the involvement of specialist PC services can modify the socio-economic effect on the PLC [22]. In our study, the previous involvement of an outpatient PC service did indeed lower the odds of dying in the hospital, and this was trued to regardless of socio-economic status. While it was expected that patients admitted from the ED/ICU had higher odds of dying on hospital wards, identifying a DNR order as a determinant for a hospital ward to be the PLC is counterintuitive. On the one hand, a DNR order, which is often part of advance care directives or advanced care planning (ACP) discussions, can be seen as evidence that a patient wants less aggressive care and agrees to be admitted to the APCU rather than other wards; on the other hand, a DNR order can be the result of a medical decision by the physician team, for example, when it is deemed unlikely that a patient will profit from a possible resuscitation. Results from the literature examining the effects of a DNR status on the aggressiveness on patient care remain inconclusive [23, 24].

This is the first study examining PLC and its socio-economic determinants for palliative care patients treated at an APCU at a CCC in Switzerland. Most data was available for all patients under study and socio-economic variables were comprehensively assessed. Shortcomings of this study stem from its single-institution character and retrospective nature. No data was available to assess whether our APCU is representative of other APCUs in Switzerland or other CCC in Europe. Moreover, for about one fifth of patients, the highest level of education remained unknown. The study also does not allow conclusions regarding socio-economic differences when it comes to the general availability of palliative care services. A population-based assessment could help circumvent these limitations in the future. By nature of this study, our findings are not generalizable to other palliative patient populations and those who never made it onto the palliative care wards in the first place.

In conclusion, more than half of patients admitted for end-of-life care died on the APCU. The proportion of patients admitted from home was 26%, while only 21% of patients returned home after their hospital stay. Socio-economic factors did not determine place of last care, yet the previous involvement of an outpatient palliative care service was a lever to facilitate dying at home.

### Electronic supplementary material

Below is the link to the electronic supplementary material.

Supplementary Table 1: STROBE checklist. Description of data: Summary of the data and manuscript according to the criteria set out in the STROBE checklist for the publication of retrospective observational studies.

## Data Availability

The datasets generated and/or analyzed during the current study are not publicly available due to confidentiality and data privacy, but are available in anonymized form from the corresponding author on reasonable request.

## Code Availability

Not applicable for this publication.

## Abbreviations

APCU: Acute palliative care unit
CCC: Comprehensive cancer center
CI: Confidence interval
DNR: Do not resuscitate
ED: Emergency department
EMR: Electronic medical records
EoL: End-of-life
GDP: Gross domestic product
ICU: Intensive care unit
LoS: Length of stay
OR: Odds ratio
PLC: Place of last care
USZ: University Hospital Zurich
