# Supplementary material for: Socio-economic determinants for the place of last care: results from the acute palliative care unit of a large comprehensive cancer center in a high-income country in Europe
Source: BMC Palliat Care. 2023 Aug 8;22:114. doi: 10.1186/s12904-023-01240-2 (PMC10408184; doi:10.1186/s12904-023-01240-2)
Supplement: Supplementary file 1 — Supplementary Table 1: STROBE checklist. Description of data: Summary of the data and manuscript according to the criteria set out in the STROBE checklist for the publication of retrospective observational studies. [file 12904_2023_1240_MOESM1_ESM.docx]

Supplementary Table 1: STROBE checklist

|  | Item No. | Recommendation | Relevant text from manuscript |
| --- | --- | --- | --- |
| **Title and abstract** | 1 | (a) Indicate the study’s design with a commonly used term in the title or the abstract | Socio-economic Determinants for the Place of Last Care: Results from a Cohort Study at an Acute Palliative Care Unit of a Large Comprehensive Cancer Center in a High-Income Country in Europe |
|  |  | (b) Provide in the abstract an informative and balanced summary of what was done and what was found | The aim of this single-center retrospective study was to investigate place of last care and selected socio-economic determinants for the subgroup of patients who required hospitalization on an acute palliative care unit of a large comprehensive cancer center in Switzerland. More than half of patients died on inpatient wards in 2019; socio-economic determinants were not associated with place of last care. |
| Introduction | |  |  |
| Background/rationale | 2 | Explain the scientific background and rationale for the investigation being reported | Last place of care (LPC) is an important aspect of end-of-life (EoL) care. Catering to patients’ preferences with respect to LPC is increasingly regarded as a quality marker for EoL care. LPC is influenced by many factors, including the structure of the social welfare and healthcare systems, cultural and personal beliefs and preferences, as well as the clinical disease reality with its associated symptoms. In high-income countries, the majority of people would like to die at home. Yet a large proportion of patients continues to die in hospitals and long-term care facilities. While in low- and middle-income countries, a larger proportion of patients is estimated to die at home, this is often due to a lack of adequate healthcare resources and against the backdrop of the insufficient alleviation of suffering. |
| Objectives | 3 | State specific objectives, including any prespecified hypotheses | The aim of this study is therefore to investigate LPC and selected socio-economic determinants for the subgroup of patients who required hospitalization on the acute palliative care unit (APCU) of a large comprehensive cancer center (CCC) in Switzerland, which serves as an exemplary high-income country with a high-quality healthcare system in the heart of Europe. |
| Methods | |  |  |
| Study design | 4 | Present key elements of study design early in the paper | This study was conceptualized as a single-center observational cohort study, which was conducted at the Radiation Oncology Department of the University Hospital Zurich (USZ). The APCU of our department is integral part of the university hospital’s CCC, from which patients are referred. The APCU is also open to non-oncological patients. |
| Setting | 5 | Describe the setting, locations, and relevant dates, including periods of recruitment, exposure, follow-up, and data collection |  |
| Participants | 6 | (a) Cohort study—Give the eligibility criteria, and the sources and methods of selection of participants. Describe methods of follow-up  Case-control study—Give the eligibility criteria, and the sources and methods of case ascertainment and control selection. Give the rationale for the choice of cases and controls  Cross-sectional study—Give the eligibility criteria, and the sources and methods of selection of participants |  |
|  |  | (b) Cohort study—For matched studies, give matching criteria and number of exposed and unexposed  Case-control study—For matched studies, give matching criteria and the number of controls per case | Not applicable. |
| Variables | 7 | Clearly define all outcomes, exposures, predictors, potential confounders, and effect modifiers. Give diagnostic criteria, if applicable | The study was conceptualized to analyze PLC and its socio-economic determinants. PLC was defined as the location, where patients spent at least the last couple of days of their lives. Hospital, acute care facility, rehabilitation facility, hospice and home were chosen as PLC categories. For patients who did not decease on the APCU and were discharged, the PLC was established via a thorough review of data and medical files, which emanated from external caregivers. The following socio-economic variables were assessed: Gender, nationality, confession, health insurance plan, highest level of completed education, general living situation (urban/rural), social living situation pre-admission, and next relative. Selected factors pertaining to individual preferences or aspects of EoL care were also assessed. |
| Data sources/ measurement | 8* | For each variable of interest, give sources of data and details of methods of assessment (measurement). Describe comparability of assessment methods if there is more than one group |  |
| Bias | 9 | Describe any efforts to address potential sources of bias | Biases inherent to retrospective case series could not be circumvented. |
| Study size | 10 | Explain how the study size was arrived at | All adult patients hospitalized on our APCU from January to December 2019 qualified for this study. |
| Quantitative variables | 11 | Explain how quantitative variables were handled in the analyses. If applicable, describe which groupings were chosen and why | Manually extracted data was assembled in the spreadsheet program Microsoft© Excel© (version v.16). Descriptive summary statistics were computed for all variables under study. Uni- and multivariable logistic regression analysis was used to identify determinants for the PLC. Statistical significance was set at <0.05, as is common in the medical literature. Statistical analysis was conducted by one researcher (SMC) and quality-checked by another researcher (CH). The statistical software package STATA (v16.1) was used to conduct all quantitative analysis. |
| Statistical methods | 12 | (a) Describe all statistical methods, including those used to control for confounding |  |
|  |  | (b) Describe any methods used to examine subgroups and interactions |  |
|  |  | (c) Explain how missing data were addressed |  |
|  |  | (d) Cohort study—If applicable, explain how loss to follow-up was addressed  Case-control study—If applicable, explain how matching of cases and controls was addressed  Cross-sectional study—If applicable, describe analytical methods taking account of sampling strategy | Not applicable. |
|  |  | (e) Describe any sensitivity analyses | Not applicable. |
| **Results** |  |  |  |
| Participants | 13* | (a) Report numbers of individuals at each stage of study—eg numbers potentially eligible, examined for eligibility, confirmed eligible, included in the study, completing follow-up, and analyzed | All patients hospitalized on the palliative care wards, a total of 377 patients, was included into the analysis. As data was available for the overwhelming majority of all patients (except for highest level of education), no patients had to be excluded from the analyses. |
|  |  | (b) Give reasons for non-participation at each stage |  |
|  |  | (c) Consider use of a flow diagram | Not included into this study, as patient recruitment for this study was simple. |
| Descriptive data | 14* | (a) Give characteristics of study participants (eg demographic, clinical, social) and information on exposures and potential confounders | Median age of the 377 patients under study was 71 (interquartile range (IQR), 59–81). Forty-four (n=167) percent of patients were female. The large majority (80%; n=300) were Swiss. Slightly less than two thirds of patients (60%; n=226) were of Christian faith. Almost 80% (n=297) of patients had public health insurance. The highest level of completed education was high school or tertiary education for 77% (n=287) patients, while for 23% (n=88) of patients, education status remained unknown. For 80% (n=300) of patients, cancer was the primary diagnosis. |
|  |  | (b) Indicate number of participants with missing data for each variable of interest | For highest level of education, for about a fifth of patients, no data was available in the electronic medical records. |
|  |  | (c) Cohort study—Summarize follow-up time (eg, average and total amount) | Patients were not followed-up, as this is an inpatient study. The median length of stay (LoS) on the palliative care wards for all patients was 11 (5–17) days. |
| Outcome data | 15* | Cohort study—Report numbers of outcome events or summary measures over time | By nature of the study, all patients received inpatient specialist PC (100%; n=377). Prior to hospital admission, the General Practioner (GP) was closely involved in the coordination or direct patient care in 74% (n=279) of cases. Post-discharge, the outpatient specialist PC was employed in slightly more than a quarter of patients (27%; n=103), while the outpatient home care service was utilized by 36% (n=134) of all patients. Sixty percent (n=224) of patients had advanced care directives, 55% (n=206) had named a patient representative. While the ICU status was affirmative for less than ten percent of patients (9%; n=32), 97% (n=363) of patients had an affirmative do-not-resuscitate (DNR) status (Table 3). |
|  |  | Case-control study—Report numbers in each exposure category, or summary measures of exposure | Not applicable. |
|  |  | Cross-sectional study—Report numbers of outcome events or summary measures | Not applicable. |
| Main results | 16 | (a) Give unadjusted estimates and, if applicable, confounder-adjusted estimates and their precision (eg, 95% confidence interval). Make clear which confounders were adjusted for and why they were included | On univariable logistic regression analysis, ten variables were significantly associated with the inpatient setting being the PLC. Age older than or equal to 70 years (Odds ratio (OR), 0.521 (95% confidence interval (CI), 0.312–0.872; p-value <0.05), LoS of 10 days or more (OR, 1.773; 95% CI, 1.058–2.972; p-value <0.05), admission from ED or ICU (OR, 10.991; 95% CI, 2.630–45.931; p-value <0.001), and cancer diagnosis (OR, 3.874; 95% CI, 1.617–9.282; p-value <0.05) were positively associated with “hospital” as PLC. Additionally, a partner as next relative (OR, 0.580; 95% CI, 0.338–0.994; p-value <0.05), outpatient PC involvement pre-admission (OR, 0.083; 0.047–0.149; p-value <0.001), no outpatient home care involvement pre-admission (OR, 0.301; 95% CI, 0.180–0.505; p-value <0.001), advance care directives (OR, 0.549; 95% CI, 0.320–0.942; <0.05), negative ICU status (OR, 0.215; 95% CI, 0.102–0.453; p-value <0.001), and DNR order (OR, 0.036; 95% CI, 0.008–0.166; p-value <0.001) were also positively associated with hospital as LPC. On multivariable logistic regression analysis, the effect of three variables persisted, namely admission from ED/ICU (OR, 24.565; 95% CI, 2.095– 288.023; p-value <0.05), previous involvement of outpatient PC service (OR, 0.105; 95% CI, 0.052–0.213; p-value <0.001), and DNR order (OR, 0.012; 95% CI, 0.000–0.171; p-value <0.001). |
|  |  | (b) Report category boundaries when continuous variables were categorized |  |
|  |  | (c) If relevant, consider translating estimates of relative risk into absolute risk for a meaningful time period |  |
| Other analyses | 17 | Report other analyses done—eg analyses of subgroups and interactions, and sensitivity analyses | Not applicable. |
| **Discussion** |  |  |  |
| Key results | 18 | Summarize key results with reference to study objectives | In summary, 377 patients with a median age of 71 years, 44% of which were female, were hospitalized on the APCU for a median of 11 days in 2019. Most patients were Swiss (80%), Christian (60%) and had completed high school or tertiary education (77%). APCU was the PLC for 54% of patients, 26% were transferred to other facilities and 20% were discharged home. Admission from ED/ICU, no previous involvement of an outpatient PC service, and an active resuscitation order were positively associated with dying in the inpatient setting. Gender, nationality, religion, health insurance plan, and highest level of completed education were no determinants for PLC. |
| Limitations | 19 | Discuss limitations of the study, taking into account sources of potential bias or imprecision. Discuss both direction and magnitude of any potential bias | Shortcomings of this study stem from its single-institution character and retrospective nature. No data was available to assess whether our APCU is representative of other APCUs in Switzerland or other CCC in Europe. Moreover, for about one fifth of patients, the highest level of education remained unknown. The study also does not allow conclusions regarding socio-economic differences when it comes to the general availability of palliative care services. A population-based assessment could help circumvent these limitations in the future. |
| Interpretation | 20 | Give a cautious overall interpretation of results considering objectives, limitations, multiplicity of analyses, results from similar studies, and other relevant evidence | In conclusion, more than half of patients admitted for end-of-life care died on the APCU. The proportion of patients admitted from home was 26%, while only 21% of patients returned home after their hospital stay. Socio-economic factors did not determine place of last care, yet the previous involvement of an outpatient palliative care service was a lever to facilitate dying at home. |
| Generalizability | 21 | Discuss the generalizability (external validity) of the study results | By nature of this study, our findings are not generalizable to other palliative patient populations and those who never made it onto the palliative care wards in the first place. |
| Other information |  |  |  |
| Funding | 22 | Give the source of funding and the role of the funders for the present study and, if applicable, for the original study on which the present article is based | No funding was received explicitly for this project. SMC is on research leave and lead this project during this time. |
